# Evaluation of left ventricular diastolic function in patients operated for aortic stenosis

**DOI:** 10.1371/journal.pone.0263824

**Published:** 2022-02-25

**Authors:** Henrik Hultkvist, Eva Nylander, Éva Tamás, Rolf Svedjeholm, Jan Engvall, Jonas Holm, Eva Maret, Farkas Vánky

**Affiliations:** 1 Department of Cardiothoracic and Vascular Surgery, Heart Centre, Linköping University Hospital, Linköping, Sweden; 2 Department of Clinical Physiology and Department of Health, Medicine and Caring Sciences, Linköping University, Linköping, Sweden; 3 Center for Medical Image Science and Visualization (CMIV), Linköping University, Linköping, Sweden; 4 Department of Clinical Physiology, Karolinska University Hospital and Karolinska Institutet, Stockholm, Sweden; Scuola Superiore Sant’Anna, ITALY

## Abstract

**Background:**

Left ventricular diastolic dysfunction is common in patients with aortic valve stenosis (AS) and reportedly affects prognosis after surgical aortic valve replacement (SAVR). Here we investigated whether and how diastolic function (assessed following the most recent guidelines) was affected by SAVR, and whether preoperative diastolic function affected postoperative outcome. We also examined whether long-term mortality was associated with preoperative NT-proBNP and postoperative heart failure (PHF).

**Methods:**

We performed a prospective observational study of 273 patients with AS who underwent AVR with or without concomitant coronary artery bypass surgery. Of these patients, 247 were eligible for assessment of left ventricular (LV) filling pressure. Preoperatively and at the 6-month postoperative follow-up, we measured N-terminal pro-B type natriuretic peptide (NT-proBNP) in serum and assessed diastolic function with Doppler echocardiography. PHF was diagnosed using prespecified criteria. Multivariable logistic regression was performed to explore variables associated with high LV filling pressure. Cox regression was performed to explore variables associated with mortality, accounting for timeto-event.

**Results:**

At the time of surgery, 22% (n = 54) of patients had diastolic dysfunction expressed as high LV filling pressure. Of these 54 patients, 27 (50%) showed postoperative diastolic function improvement. Among the 193 patients with preoperative low LV filling pressure, 24 (12%) showed postoperative diastolic function deterioration. Increased long-term mortality was associated with PHF and high preoperative NT-proBNP, but not with preoperative or postoperative diastolic dysfunction. Cox regression revealed the following independent risk factors for long-term mortality: diabetes, renal dysfunction, preoperative NT-proBNP>960 ng/L, age, and male gender.

**Conclusions:**

Surgery for aortic stenosis improved diastolic function in patients with high LV filling pressure in 50% of the patients. Our results could not confirm the previously suggested role of diastolic dysfunction as a marker for poor long-term survival after SAVR. Our findings showed that both PHF and high preoperative NT-proBNP were associated with long-term mortality.

## Introduction

Hemodynamically significant aortic valve stenosis (AS) results in concentric left ventricular (LV) hypertrophy caused by efforts to maintain normal wall tension. LV hypertrophy affects LV diastolic function and is often accompanied by increased diffuse interstitial fibrosis in advanced AS [1]. The diastole can be divided into three phases: relaxation with rapid ventricle filling, diastasis, and end-diastole with atrial contraction. Initial LV filling is mainly influenced by relaxation, which is an active calcium-dependent process, while LV filling towards the end of diastole relies more on LV compliance. Early milder diastolic dysfunction is often characterized by abnormal relaxation, but normal filling pressure (at least at rest), while more severe diastolic dysfunction involves reduced compliance, resulting in high LV filling pressure.

Diastolic dysfunction is reportedly common after aortic valve replacement (AVR), and is associated with mortality [2, 3]. It has also been proposed that diastolic dysfunction may lead to heart failure many years after surgery [2, 4]. Conflicting data have been reported regarding whether surgical AVR (SAVR) actually improves diastolic function [5].

Echocardiography is frequently used to evaluate diastolic function. Upon the development of blood flow Doppler, this method became more convenient and widely used.

The later introduction of tissue Doppler and other modalities brought additional options for diastolic function characterization [6]. Different generations of guidelines all recommend the use of combinations of parameters for the characterization of diastolic function. The most recent guidelines were published in 2016 by the European Association of Cardiovascular Imaging (EACVI) and the American Society of Echocardiography [7].

B-type natriuretic peptide (BNP) is an endogenous hormone that counteracts some symptoms of heart failure—affecting diuresis and vasodilation, having anti-hypertrophy and anti-fibrosis properties, and exerting inhibitory effects on the sympathetic nervous system and renin-angiotensin-aldosterone system [2]. The amino-terminal fragment of BNP’s prohormone, NT-proBNP, is released into circulation from myocytes in response to myocyte stretch, increased wall tension, and ischemia [3, 4], and is a well-established biomarker of heart failure [5]. We previously described the dynamics of NT-proBNP in aortic valve surgery, and its association with 1-year mortality [8]. The clinical manifestation of postoperative cardiac dysfunction, defined as postoperative heart failure (PHF), is also associated with poor outcome after AVR [9, 10].

In the present study, we aimed to examine how surgery for aortic stenosis impacted LV diastolic function, using an adapted version of the most recent guidelines. We further evaluated how outcome after aortic valve surgery for AS was impacted by pre- and postoperative high LV filling pressure, NT-proBNP level, and PHF. Our hypotheses were that diastolic LV function improves after surgery for AS, and that pre- and postoperative high LV filling pressures negatively impact post-SAVR outcome, alone or in combination with elevated NT-proBNP and PHF.

## Methods

This prospective longitudinal observation study included 273 patients scheduled for aortic valve surgery due to aortic stenosis between June 2008 and January 2013. Patients were recruited from the south-east region of Sweden, and surgery was performed at Linköping University Hospital—the only tertiary care hospital performing open heart surgery, which serves a population of over one million people. Exclusion criteria were emergency operations, active endocarditis, double-valve procedures, and inability to provide written informed consent. Patients with coronary artery disease or undergoing ascending aorta replacement without circulatory arrest were not excluded.

The main protocol included obtaining blood samples for NT-proBNP measurement and performing a detailed echocardiographic evaluation of diastolic LV function before surgery and at 6 months post-surgery. Clinical data were prospectively registered in our clinical database (Carath; Fujitsu, Tokyo, Japan) and mortality records were retrieved from the Swedish Civil registry.

### Definitions

The clinical manifestation of low cardiac output syndrome (LCOS) with post-cardiotomy cardiogenic shock was evident at weaning from cardio-pulmonary bypass (CPB) or during the early hours after surgery. We also included cases not fulfilling criteria for cardiogenic shock and defined postoperative heart failure (PHF) as a hemodynamic state at which cardiac output does not meet systemic demand without supportive measures, other than correction of volume or vascular resistance. This hemodynamic state was assessed using previously reported mixed venous oxygen criteria [11]. Supportive measures and treatment comprised the use of an intra-aortic balloon pump or ventricular assist device, or infusion of one or more inotropes for over 30 minutes at the following dosages: epinephrine, ≥0.05–0.3 μg/kg body weight per minute; milrinone, ≥0.375 μg/kg body weight per minute; dopamine, ≥4 μg/kg body weight per minute; dobutamine, ≥4 μg/kg body weight per minute; and levosimendan 0.1 (0.05–0.2)μg/kg body weight in 24 hours. Perioperative myocardial infarction (PMI) was defined as a sustained elevation of troponin T to ≥2000 ng/L on or after the third postoperative day [12].

### Echocardiography

Echocardiographic examination was performed by experienced technicians on the day before surgery and at the 6-month postoperative follow-up. Preoperative examination was conducted using Vivid V7 and E9 ultrasound systems (GE Ultrasound, Horten, Norway). For postoperative examination, some patients were additionally examined using Philips or Siemens systems. All echocardiograms were evaluated and double-checked by three senior experienced physicians.

Standard 2-dimensional and Doppler echocardiography and measurements were performed in accordance with guidelines [7, 13]. We evaluated diastolic function using a guideline-based automated decision algorithm (https://liu.se/en/research/left-ventricularhttps://liu.se/en/research/left-ventricular-diastolic-function-decision-supportdiastolic-function-decision-support), which accounted for age-adjusted normal ranges [14]. Briefly, the evaluation was primarily based on mitral E/A ratio, pulmonary venous systolic and diastolic flow velocities, E/eʹ (mean of septal and lateral eʹ) and left atrial size (left atrial end systolic area in the 4-chamber view, indexed to BSA; LAAi). If a patient had contradictory or otherwise unclassifiable measurements, an individualized evaluation was performed with consideration of mitral flow deceleration time, isovolumetric relaxation time, and tricuspid regurgitation velocity. General instructions regarding the applicability of diastolic function criteria described in the guidelines were adhered to, i.e., diastolic function was not evaluated in patients with atrial fibrillation, neither in patients with significant mitral annular calcification nor in cases of insufficient quality of the registrations.

Left ventricular diastolic function (LVDF) was classified into four categories (normal or Grade I–III dysfunction) according to guidelines. Normal and Grade I dysfunction are considered to be associated with low LV filling pressure, and Grade II and III dysfunction with high LV filling pressure. All data were digitally stored using EchoPAC version BT 11 (GE Healthcare, Horton, Norway). Patients with atrial fibrillation at echocardiographic evaluation were excluded from analysis of diastolic function. For each case, systolic LV function was semiquantitatively evaluated by two observers, using a 4-degree scale (normal, mildly abnormal, moderately abnormal, and severely abnormal), corresponding to the EF categories recommended by Lang et al. [13].

### NT-proBNP

Blood samples for plasma NT-proBNP analysis were obtained on the day before surgery and at the 6-month follow-up. Samples were collected in lithium heparin tubes and analyzed within 3 hours. Plasma levels of NT-proBNP were measured using an electro-chemo luminescence immunoassay with a Roche Elecsys 2010 automated device (Roche Diagnostics, Basel, Switzerland). The results were blinded prior to data analyses. Analyses were performed in one core laboratory. The assay had an effective measurement range of 5–35 000 ng/L, and inter-assay variability or coefficient of variation (CV) was <4% for all intervals.

### Clinical management

All patients underwent surgery via full sternotomy and involving standard use of cardiopulmonary bypass with normal or moderate hypothermia and aortic occlusion. Diluted blood cardioplegia (one part blood and four parts crystalloid cardioplegia, supplemented with procaine hydrochloride) was used with an antegrade or combined antegrade and retrograde delivery system (Plegisol™, Abbot, IL, USA).

### Statistics

Continuous data are presented as median with interquartile range (IQR), and categorical variables as number and percentage (%). For between-group comparisons, the Mann-Whitney U test was used for continuous data, and the X^2^ test and Fisher’s exact test for dichotomous data. Pairwise comparisons were performed using the Wilcoxon matched pairs signed rank sum test. Multivariable logistic regression was applied to explore variables associated with high filling pressure, and Cox regression was used to explore variables associated with mortality, accounting for time-to-event. Variables were selected a priori, accounting for confounders as described by Stralen et al. [15]. Variables were tested in the final model if *P*<0.1. The finite number of terms in the final model was parsimoniously chosen according to the limited number of events. We performed receiver operating characteristic (ROC) analyses to evaluate the discrimination of preoperative NT-proBNP on 5-year mortality, and Youden’s index was used to determine the best cut-off point (NT-proBNP>960 ng/L) with regards to specificity and sensitivity. Survival curves were calculated using the Kaplan-Meier method, and the log-rank test was performed to investigate between-group differences in survival times. Statistical analyses were performed using NCSS 12 Statistical Software, 2018 (NCSS, LLC. Kaysville, Utah, USA, ncss.com/software/ncss) and Statistica v.13.2 (StatSoft, Tulsa, Oklahoma, USA).

### Ethics

This study was approved by The Regional Ethics Review Board (M 198–07, T 126–08, 2012/422-32), and patients were included only after providing written informed consent, in accordance with the World Medical Association’s Helsinki declaration on ethical principles for medical research.

## Results

A complete echocardiographic evaluation with regards to diastolic function was possible in 247 of 273 patients. Patients were divided into two groups based on preoperative diastolic function: patients with signs of low LV filling pressure (n = 193/247; 78%), and those fulfilling criteria for high LV filling pressure (n = 54/247; 22%). Patients with high LV filling pressure were significantly older, presented with higher EuroSCORE II, and presented with more comorbidities. Table 1 shows patients’ baseline characteristics and preoperative data, and Table 2 summarizes the perioperative data and adverse events.

**Table 1:** Patients’ baseline characteristics^a^.

Variable	Preoperative low LV filling pressure (n = 193)	Preoperative high LV filling pressure (n = 54)	*P* value
Age, years	70 [65–76]	75 [67–80]	0.01
Sex, female	89 (46%)	24 (44%)	0.82
BMI, kg/m^2^	27 [24–30]	27 [25–30]	0.36
BSA, m^2^	1.9 [1.8–2.1]	1.9 [1.8–2.1]	0.88
Hypertension	99 (51%)	34 (63%)	0.13
Diabetes mellitus	28 (15%)	13 (24%)	0.09
(insulin or oral treatment)			
Angina pectoris	51 (26%)	23 (43%)	0.02
CHF	12/191 (6%)	15/53 (28%)	<0.001
EuroSCORE II	1.6 [1.1–2.7]	3.1 [1.6–5.4]	<0.001
NYHA III/IV	99 (52%)	36 (67%)	0.04
Coronary artery disease	84 (44%)	40 (75%)	<0.001
Prior myocardial infarction	11 (6%)	8 (15%)	0.03
Diastolic function grade/normal	160 (83%)	-	-
Diastolic function grade/ I	33 (17%)	-	-
Diastolic function grade/II	-	19 (30%)	-
Diastolic function grade/III	-	35 (65%)	-
***Medications***			
ACE inhibitors	46 (30%), n = 154	14 (36%), n = 39	0.56
Calcium channel blockers	30 (20%), n = 153	7 (18%), n = 39	>0.99
Diuretics	38 (25%), n = 154	9 (23%), n = 39	>0.99
Beta blockers	76 (49%), n = 154	33 (85%), n = 39	<0.001
**Biochemistry**			
P-Cystatin C, mg/L	1.1 [1.0–1.3], n = 184	1.3 [1.0–1.4], n = 53	0.003
P-Creatinine clearance, mL/min/m^2^	74 [61–92]	64 [48–81]	0.006
Blood hemoglobin, g/L	140 [133–148]	132 [123–146]	0.01
NT-proBNP, ng/L	320 [170–775], n = 189	1100 [510–3030], n = 53	<0.001
NT-proBNP>960 ng/L	32/189 (17%)	30/53 (57%)	<0.001
**Echocardiography**			
***Systolic LVF***			
Normal/mild dysfunction	186/191 (97%)	45/53 (85%)	0.002
LV mass index, g/m^2^	125 [101–146], n = 158	137 [109–172], n = 44	0.05
• >95 (female)^b^	52/72 (72%)	16/18 (89%)	0.22
• >115 (male)^c^	65/86 (76%)	20/26 (77%)	0.89
***Diastolic LVF***			
E/A	0.8 [0.7–0.9], n = 193	1.6 [1.2–2.1], n = 54	
E/eʹ	8 [6–10], n = 191	8 [6.5–9.5], n = 54	
• >14^d^	96/193 (50%)	43/54 (80%)	
LAA index, cm^2^/m^2^	11.3 [9.8–12.9], n = 193	12.9 [11.2–15.1], n = 54	
•>13^e^	45/193 (23%)	26/54 (48%)	
PV s/PV d^f^	1.5 [1.3–1.8], n = 192	0.8 [0.5–1], n = 54	
***Aortic valve***			
EOA, cm^2^	0.6 [0.5–0.8], n = 192	0.6 [0.5–0.8], n = 54	0.10
iEOA, cm^2^/m^2^	0.3 [0.3–0.4], n = 192	0.3 [0.2–0.4], n = 54	0.12
Mean pressure gradient, mmHg	55 [44–68], n = 188	49 [36–69], n = 53	0.11

^a^ Data are presented as median [IQR] or number (%).

BMI, body mass index; CHF, congestive heart failure; EuroSCORE II, European system for cardiac operative risk evaluation; NYHA, New York Heart Association functional classification; LVF, left ventricular function; LV mass index, left ventricular mass/BSA; BSA, body surface area

^b^, upper limit values for normal LV mass for females

^c^, upper limit values for normal LV mass for males; E/A, early diastolic mitral flow velocity/late diastolic mitral flow velocity; E/eʹ, early diastolic mitral flow velocity/early diastolic myocardial velocity

^d^, reference value for E/eʹ indicating high filling pressure (Nagueh et al., J Am Soc Echocardiogr, 2016); LAA index, left atrial area/BSA

^e^, reference value for enlarged left atrium (Kou et al., Eur Heart J Cardiovasc Imaging, 2014;15(6):680–90)

^f^, pulmonary vein systolic flow velocity/pulmonary vein diastolic flow velocity; EOA, effective orifice area; iEOA, EOA/BSA.

**Table 2:** Perioperative data and adverse events.

Variable	Preoperative low LV filling pressure (n = 193)	Preoperative high LV filling pressure (n = 54)	*P* value
Concomitant CABG	48 (25%)	21 (39%)	0.04
Elective surgery	189 (98%)	54 (100%)	0.58
Redo surgery	3 (1.6%)	1 (1.8%)	>0.99
Cross-clamp time min	70 [61–88]	70 [62–87]	0.87
ECC time, min	96 [83–117]	101 [85–126]	0.42
***Valve type***			
Mechanical	99 (51%)	19 (35%)	0.04
Biological	94 (49%)	35 (65%)	0.04
***Outcome***	3 [2–5]	4 [3–8]	0.04
Time on ventilator, hours			
Time in ICU, hours	21 [17–22]	22 [21–24]	0.02
CK-MB day 1	19 [14–27]	18 [12–27], n = 52	0.59
P-Creatinine, maximum, μmol/L	87 [70–106], n = 192	106 [83–134]	<0.001
P-Creatinine, at discharge, μmol/L	81 [67–99], n = 192	88 [76–119]	0.003
P-Cystatin C, Day 3, mg/L	1.3 [1.1–1.6], n = 180	1.6 [1.3–1.9]	0.002
Postoperative AF	84/193 (44%)	24/53 (45%)	0.72
Stroke	2/193 (1.0%)	0	>0.99
PMI	7/193 (3.6%)	2 (3.8%)	>0.99
PHF	14 (7.3%)	12 (22.2%)	0.002
Reoperation valve failure or PVL	0	0	
Reoperation DSWI	0	1 (1.9%)	0.22
30-day mortality	0	1 (1.9%)	>0.22
1-year mortality	4 (2.1%)	2 (3.7%)	>0.61
Long-term mortality*	38 (20%)	14 (26%)	0.32
**Echocardiographic data and NT-proBNP at 6-month follow-up**			
***Systolic LVF***			
Normal/mild	176/181 (97%)	40/49 (82%)	<0.001
dysfunction			
VTI (LVOT)˚, cm	22 [18–25], n = 179	22 [18–26], n = 47	>0.99
LV mass index, g/m^2^	96 [83–109], n = 135	109 [89–129], n = 42	0.005
• >95 (female)^b^	20/58 (34%)	10/17 (59%)	0.07
• >115 (male)^c^	16/77 (21%)	13/25 (52%)	0.003
LV mass index regression*, g/m^2^	24 [4–41], n = 114	32 [5–47], n = 37	0.40
***Aortic valve***			
EOA, cm^2^	1.4 [1.2–1.6], n = 177	1.3 [1.0–1.4], n = 45	0.01
iEOA, cm^2^/m^2^	0.7 [0.6–0.8], n = 177	0.6 [0.5–0.7], n = 45	0.003
PPM	140/193 (73%)	38/54 (70%)	0.75
Severe PPM	58/193 (30%)	26/54 (48%)	0.01
Mean pressure gradient, mmHg	13 [11–16], n = 179	14 [10–18], n = 49	0.52
***NT-proBNP***			
NT-proBNP, ng/L	270 [150–490], n = 172	760 [370–1090], n = 45	<0.001
NT-proBNP>960 ng/L**	32/189 (17%)	30/53 (57%)	<0.001

^a^ Data are presented as median [IQR] or number (%).

AF, atrial fibrillation; CABG, coronary artery bypass surgery; ECC, extra corporeal circulation; ICU, intensive care unit; PMI, perioperative myocardial infarction; PHF, postoperative heart failure; PVL, paravalvular leakage; DSWI, deep sternal wound infection; *, median follow-up of 7.1 years (4.1–9.1 years); LVF, left ventricular function

^b^, upper limit values for normal LV mass for females

^c^, upper limit values for normal LV mass for males; E/A, early diastolic mitral flow velocity/late diastolic mitral flow velocity; E/eʹ, early diastolic mitral flow velocity/early diastolic myocardial velocity

^d^, reference value for E/eʹ indicating high filling pressure (Nagueh et al., J Am Soc Echocardiogr, 2016); LAA index, left atrial area/BSA

^e^, reference value for enlarged left atrium (Kou et al., Eur Heart J Cardiovasc Imaging, 2014;15(6):680–90)

^f^, pulmonary vein systolic flow velocity/pulmonary vein diastolic flow velocity; EOA, effective orifice area; PPM, prostheses-patient mismatch; *, preoperative LV mass index-postoperative LV mass index

**, best cut-off point for preoperative NT-proBNP with regards to discriminating 5-year mortality.

### Diastolic LV function

**Fig 1:**
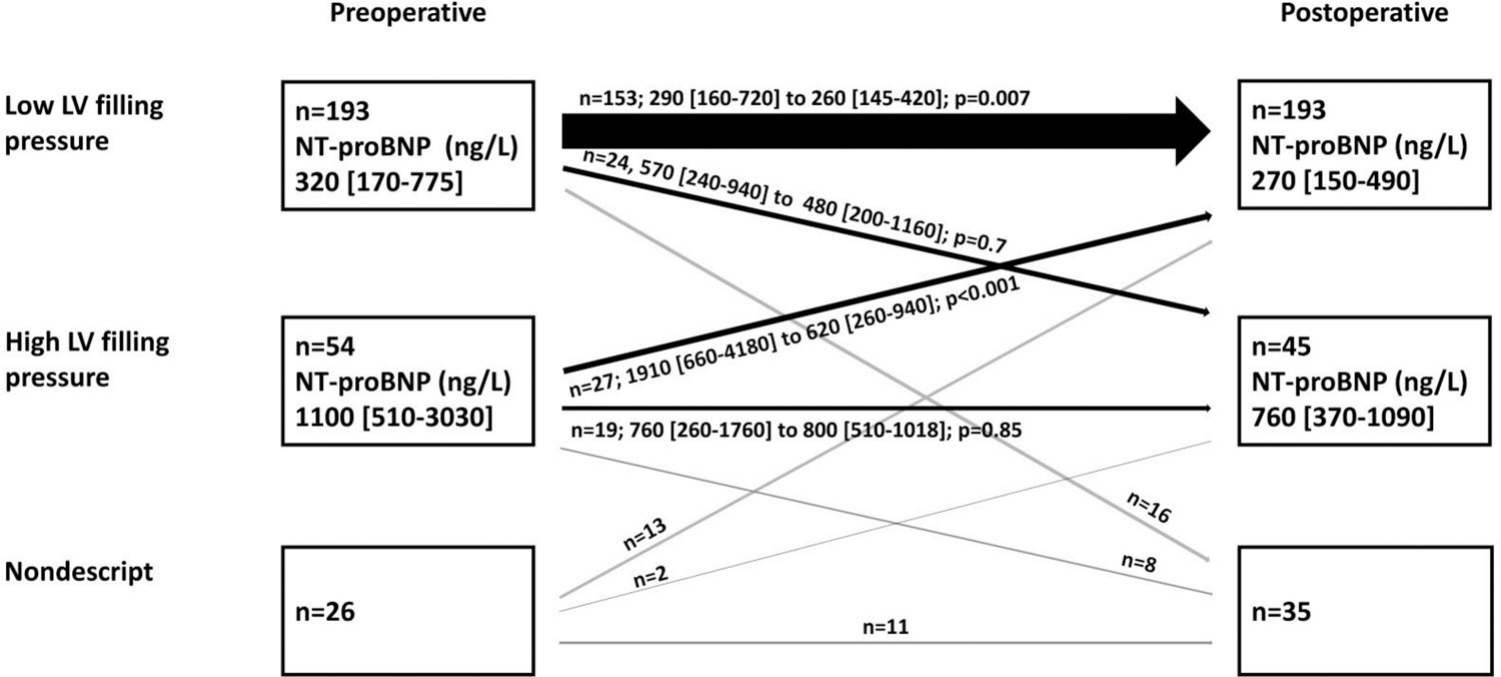
Diastolic function categorized as low or high left ventricle (LV) filling pressure before surgery and 6 months after surgical aortic valve replacement (SAVR), along with the corresponding levels of NT-proBNP (ng/L).

Of the 54 patients with preoperative high LV filling pressure, 27 (50%) improved and showed postoperative low LV filling pressure. Among the 193 patients with preoperative low LV filling pressure, 24 (12%) deteriorated and exhibited postoperative high LV filling pressure (*P*<0.001) (Fig 1). Table 3 presents the data regarding patients who changed from preoperative low LV filling pressure to postoperative high LV filling pressure (n = 24), and those patients who maintained low LV filling pressure (n = 153). Compared to patients who maintained low LV filling pressure, those who showed diastolic function deterioration at the 6-month postoperative follow-up (n = 24) had a significantly longer time on ECC (120 min [88–132 min] vs. 94 min [91–112 min]; *P* = 0.02) and longer cross-clamp time (90 min [64–100 min] vs. 68 min [60–82 min]; *P* = 0.02), and more commonly underwent concomitant CABG procedure (10 (42%) vs. 35 (23%); *P* = 0.049) (Table 3). Table 4 presents the patients who showed improved diastolic function (n = 27) compared to those who maintained high LV filling pressure (n = 19) postoperatively.

**Table 3:** Data from patients with preoperative low filling pressure^a^.

Variable	Low preoperative LV filling pressure sustained n = 153	Low preoperative LV filling pressure deteriorated n = 24	*P* value
Age, years	70 [65–76]	70 [64–77]	0.98
Sex, female	70/153 (46%)	9/24 (38%)	0.45
BMI, kg/m^2^	27 [25–30]	26 [24–29]	0.29
BSA, m^2^	1.9 [1.8–2.1]	2.0 [1.8–2.1]	0.38
Hypertension	77/153 (50%)	13/24 (54%)	0.73
Diabetes mellitus (insulin or oral treatment)	23/153 (15%)	2/24 (8%)	0.38
Angina pectoris	40/153 (26%)	9/24 (38%)	0.25
CHF	8/151 (5%)	2/24 (8%)	0.54
EuroSCORE II	1.6 [1.1–2.7]	1.8 [1.2–3.1]	0.45
NYHA III/IV	79/153 (52%)	11/24 (46%)	0.6
Concomitant CABG	35/153 (23%)	10/24 (42%)	0.05
Elective surgery	149/153 (97%)	24/24 (100%)	>0.99
Redo surgery	2/153 (1%)	0/24 (0%)	>0.99
***Medications***			
ACE inhibitors	48 (35%), n = 139	7 (50%), n = 14	0.26
Calcium channel blockers	25 (18%), n = 139	3 (21%), n = 14	0.72
Diuretics	35 (25%), n = 139	4 (29%), n = 14	0.75
Beta blockers	99 (71%), n = 139	13 (93%), n = 14	0.11
**Biochemistry**			
P-Cystatin C, mg/L	1.4 [1–1.3], n = 145	1.1 [1–1.3], n = 23	0.71
P-Creatinine clearance, mL/min/m^2^	74 [61–91]	74 [52–106]	0.85
Blood hemoglobin, g/L	140 [133–149]	142 [136–144]	0.73
NT-proBNP, ng/L	290 [158–723], n = 150	570 [240–940], n = 23	0.09
NT-proBNP>960 ng/L	22/150 (15%)	5/23 (22%)	0.37
**Echocardiography**			
Normal/mild dysfunction	147/151 (97%)	23/24 (96%)	0.53
LV mass index, g/m^2^	126.[103–147], n = 125	119 [97–128], n = 21	0.27
• >95 (female)^b^	41/55 (759%)	6/9 (67%)	0.69
• >115 (male)^c^	54/70 (77%)	8/12 (67%)	0.47
EOA, cm^2^	0.6 [0.5–0.7], n = 152	0.7 [0.6–0.8]	0.28
iEOA, cm^2^/m^2^	0.3 [0.3–0.4], n = 152	0.3 [0.3–0.4]	0.49
Mean pressure gradient, mmHg	55 [44–68], n = 149	52 [43–62], n = 23	0.38
**Perioperative data**			
Cross-clamp time, min	68 [60–82]	90 [64–100]	0.02
ECC-time, min	94 [84–113]	120 [88–133]	0.02
Time on ventilator, hours	3 [2–5]	3 [2–5]	0.12
Time in ICU, hours	21 [17–22]	22 [18–23]	0.22
**Postoperative**			
P-Cystatin C, mg/L	1.3 [1.1–1.5], n = 143	1.3 [1.1–1.9], n = 21	0.58
Day 3			
CK-MB D1	19 [14–26]	31 [14–51]	0.04
P-Creatinine, maximum, μmol/L	87 [72–103]	97 [74–130]	0.03
P-Creatinine, at discharge, μmol/L	81 [68–95]	90 [71–107]	0.1
Postoperative AF	70/153 (46%)	9/24 (38%)	0.45
Stroke	1/153 (0.7%)	0/24 (0%)	>0.99
PMI	4/153 (2.6%)	2/24 (8%)	0.19
PHF	9/153 (6%)	2/24 (8%)	0.64
Long term mortality^p^	26/153 (17%)	7/24 (29%)	0.16
**Echocardiography 6-month follow-up**			
Normal/mild dysfunction	148/151 (98%)	22/24 (92%)	0.14
VTI (LVOT)˚, cm	22 [19–25], n = 152	22 [18–27], n = 22	0.68
LV mass index, g/m^2^	98 [84–110], n = 108	93 [82–109], n = 23	0.84
• >95 (female)	18/47 (38%)	2/9 (22%)	0.47
•>115 (male)	12/61 (20%)	4/14 (29%)	0.48
LV mass index regression*, g/m^2^	25 [1–41], n = 91	13 [4–37], n = 20	0.3
E/A	0.9 [0.8–1.1]	1.6 [1.1–2.2]	<0.001
E/eʹ	11.0 [9.0–12.5]	9.3 [7.6–13.1]	0.08
• >14	27/153 (18%)	2/24 (8%)	0.38
LAA index, cm^2^/m^2^	11.5 [10.3–12.8]	11.4 [9.8–14.8]	0.63
• >13	31/153 (20%)	8/24 (33%)	0.18
PV s/PV d^f^	1.3 [1.2–1.6]	0.8 [0.7–1]	<0.001
Velocity max, m/s	2.5 [2.2–2.7]	2.5 [2.3–2.7], n = 23	0.67
EOA, cm^2^	1.4 [1.2–1.6]	1.4 [1.1–1.5], n = 20	0.56
PPM	119/153 (78%)	18/24 (90%)	0.26
Severe PPM	50/153 (33%)	7/24 (35%)	0.84
Mean pressure gradient, mmHg	13 [11–16]	14 [12–17], n = 22	0.48
**NT-proBNP 6 month follow-up**			
NT-proBNP, ng/L	260 [143–420], n = 144	480 [200–1160], n = 23	0.003
NT-proBNP>960 ng/L**	22/150 (15%)	5/23 (22%)	0.43

^a^ Data are presented as median [IQR] or number (%).

BMI, body mass index; CHF, congestive heart failure; EuroSCORE II, European system for cardiac operative risk evaluation; NYHA, New York Heart Association functional classification; LVF, left ventricular function; LV mass index, left ventricular mass/BSA; BSA, body surface area; ^b^, upper limit values for normal LV mass for females; ^c^, upper limit values for normal LV mass for males; E/A, early diastolic filling velocity/late diastolic filling velocity; E/eʹ, early diastolic filling velocity/early diastolic myocardial velocity; ^d^, reference value for E/eʹ indicating high filling pressure (Nagueh et al., J Am Soc Echocardiogr, 2016); LAA index, left atrial area/BSA; ^e^, reference value for enlarged left atrium (Kou et al., Eur Heart J Cardiovasc Imaging, 2014;15(6):680–90); ^f^, pulmonary vein systolic flow velocity/pulmonary vein diastolic flow velocity; EAO, effective orifice area; *, preoperative LV mass index-postoperative LV mass index; ^p^, median follow-up of 7.1 years (4.1–9.1 years); **, best cut-off point for preoperative NT-proBNP with regards to discriminating 5year mortality.

**Table 4:** Data regarding patients with preoperative high filling pressure^a^.

Variable	High preoperative LV filling pressure improved (n = 27)	High preoperative LV filling pressure sustained (n = 19)	*P* value
Age, years	70 [66–78]	78 [68–80]	0.15
Sex, female	10/27 (37%)	11/19 (58%)	0.16
BMI, kg/m^2^	27 [25–31]	27 [25–30]	0.93
BSA, m^2^	1.9 [1.8–2.1]	1.9 [1.8–2.2]	0.79
Hypertension	18/27 (67%)	13/19 (68%)	0.90
Diabetes mellitus (insulin or oral treatment)	5/27 (19%)	7/19 (37%)	<0.001
Angina pectoris	9/27 (33%)	10/19 (53%)	0.19
CHF	7/26 (27%)	4/19 (21%)	0.74
EuroSCORE II	2.7 [1.2–4.3]	3.8 [1.9–5.4]	0.66
NYHA III/IV	20/27 (74%)	11/19 (58%)	0.25
Concomitant CABG	11/27 (41%)	7/19 (37%)	0.79
Elective surgery	27/27 (100%)	19/19 (100%)	-
Redo surgery	1/27 (4%)	0/19	>0.99
***Medications***			
ACE inhibitors	12 (50%), n = 24	7 (44%), n = 16	0.76
Calcium channel blockers	2 (8%), n = 24	3 (19%), n = 16	0.37
Diuretics	7 (29%), n = 24	3 (19%), n = 16	0.76
Beta blockers	22 (92%), n = 24	15 (94%), n = 16	>0.99
**Biochemistry**			
P-Cystatin C, mg/L	1.3 [1.0–1.4], n = 26	1.3 [1.2–1.4], n = 19	0.53
P-Creatinine clearance, mL/min/m^2^	64 [49–82]	69 [51–81]	0.88
Blood hemoglobin, g/L	137 [127–150]	131 [117–145]	0.47
NT-proBNP, ng/L	1910 [623–4410], n = 26	760 [260–1760]	0.035
NT-proBNP>960, ng/L	18/26 (69%)	7/19 (37%)	0.03
**Echocardiography**			
Normal/mild dysfunction	23/27 (85%)	17/18 (100%)	0.63
LV mass index, g/m^2^	143 [127–178], n = 23	111 [100–151], n = 16	0.02
• >95 (female)^b^	5/9 (56%)	4/8 (50%)	>0.99
• >115 (male)^c^	8/14 (57%)	4/8 (50%)	>0.99
EOA, cm^2^	0.5 [0.5–0.8]	0.6 [0.4–0.8]	0.60
iEAO, cm^2^/m^2^	0.3 [0.2–0.4]	0.3 [0.2–0.4]	0.60
Mean pressure gradient, mmHg	49 [36–75], n = 26	51 [38–67]	0.94
**Perioperative data**			
Cross-clamp time, min	72 [59–86]	68 [62–98]	0.66
ECC-time, min	103 [88–125]	100 [86–135]	0.91
Time on ventilator, hours	4.5 [2.8–11.7]	3.9 [2.7–7.9]	0.45
Time in ICU, hours	21.8 [20–27]	21.5 [20.8–23.5]	0.92
**Postoperative data**			
P-Cystatin C, mg/L	1.3 [1.2–1.9], n = 24	1.7 [1.3–2.0], n = 18	0.37
Day 3			
CK-MB D1	16 [13–26]	21 [13–29]	0.42
P-Creatinine, maximum, μmol/L	109 [78–126]	102 [82–137]	0.79
P-Creatinine at discharge, μmol/L	85 [73–114], n = 26	90 [72–122]	0.6
AF	7/26 (30%)	11/19 (58%)	0.04
Stroke	0	0	
PMI	1/26 (4%)	0	>0.99
PHF	7/27 (26%)	1/19 (20%)	0.12
Long term mortality^p^	8/27 (30%)	2/19 (11%)	0.16
**Echocardiography 6-month follow-up**			
Normal/mild dysfunction	21/26 (81%)	18/19 (95%)	0.22
VTI (LVOT)˚, cm	23 [18–26], n = 26	21 [19–25], n = 18	0.86
LV mass index, g/m^2^	116 [95–134], n = 22	98 [82–125], n = 17	0.13
• >95 (female)	5/7 (71%)	4/9 (44%)	0.36
• >115 (male)	8/15 (53%)	4/8 (50%)	>0.99
LV mass index regression*, g/m^2^	32 [1–47], n = 19	30 [9–44], n = 15	0.52
E/A	0.9 [0.7–1.1]	1.6 [1.2–2]	<0.001
E/é	9.8 [7.5–11.8]	10.0 [9–12.0]	0.7
• >14	3/226 (12%)	0/19	0.25
LAA-index, cm^2^/m^2^	12.8 [11.2–13.8]	13.4 [10.9–14.0]	0.76
• >13	12/27 (44%)	12/19 (63%)	0.21
PV s /PV d^f^	1.3 [1.2–1.5]	0.7 [0.6–1]	<0.001
Velocity max, m/s	2.6 [2.3–2.9]	2.5 [2.1–2.9]	0.26
EOA, cm^2^	1.3 [0.9–1.4], n = 24	1.3 [1.0–1.6], n = 18	0.52
PPM	21/27 (88%)	14/19 (74%)	0.43
Severe PPM	15/27 (63%)	8/19 (42%)	0.18
Mean pressure, mmHg	15 [11–20]	13 [8–17]	0.15
**NT-proBNP 6month follow-up**			
NT-proBNP, ng/L	620 [255–950], n = 25	800 [495–1090], n = 17	0.21
NT-proBNP>960 ng/L**	18/26 (20%)	7/19 (26–%)	0.03

^a^ Data are presented as median [IQR] or number (%).

BMI, body mass index; CHF, congestive heart failure; EuroSCORE II, European system for cardiac operative risk evaluation; NYHA, New York Heart Association functional classification; LVF, left ventricular function; LV mass index, left ventricular mass/BSA; BSA, body surface area

^b^, upper limit values for normal LV mass for females

^c^, upper limit values for normal LV mass for males; E/A, early diastolic filling velocity/late diastolic filling velocity; E/eʹ, early diastolic filling velocity/early diastolic myocardial velocity; ^d^, reference value for E/eʹ indicating high filling pressure (Nagueh et al., J Am Soc Echocardiogr, 2016).

LAA-index, left atrial area/BSA; ^e^, reference value for enlarged left atrium (Kou et al., Eur Heart J Cardiovasc Imaging, 2014;15(6):680–90)

^f^, pulmonary vein systolic flow velocity/pulmonary vein diastolic flow velocity; EAO, effective orifice area

*, preoperative LV mass index-postoperative LV mass index

^p^, median follow-up of 7.1 years (4.1–9.1 years)

**, best cut-off point for preoperative NT-proBNP with regards to discriminating 5year mortality.

In a multivariable analysis, only preoperative NT-proBNP of >960 ng/L emerged as an independent predictor of preoperative high LV filling pressure (OR, 6.4; 95% CI: 3.3–12.4; *P*<0.001). The tested variables included age, sex, body mass index (BMI), creatinine clearance, diabetes on insulin or oral treatment, hypertension, and LV mass index. Table 5 shows the variables tested for association with high LV filling pressure at the 6-month postoperative follow-up. Preoperative high LV filling pressure and aortic cross-clamp time emerged as independent predictors (Table 5).

**Table 5:** Logistic regression, univariable and multivariable analyses of postoperative high LV filling pressure.

Variable	Parameter estimate (β)	SE	Wald Χ^2^	*P* value	OR (95% CI)
Preoperative high LV filling pressure	1.5	0.37	4.02	<0.001	4.49 (2.17–9.29)
LVMI regression	−0.007	0.006	−1.03	0.3	1.00 (0.98–1.01)
Preoperative P-Creatinine clearance, mL/min/m^2^	−0.0028	0.006	−0.49	0.61	1.00 (0.99–1.01)
CCT, min	0.02	0.01	2.83	0.00468	1.02 (1.01–1.04)
NT-proBNP>960 ng/L	0.31	0.39	0.797	0.43	1.36 (0.64–2.898)
PHF	−0.26	0.65	−0.402	0.69	0.78 (0.21–2.77)
PMI	0.53	0.85	0.62	0.54	1.70 (0.32–9.1)
Severe PPM	-0.05	0.36	−0.15	0.88	0.95 (0.47–1.90)
**Final model**					
intercept	−2.17	1.68	−3.2		
CCT, min	0.02	0.08	2.7	0.006	1.03 (1.01–1.04)
Preoperative high LV filling pressure	1.52	0.38	4.0	<0.001	4.57 (2.16–9.65)

LV, left ventricular; LVMI, left ventricular mass index; CCT, cross-clamp time; ECC, extra corporeal circulation; PHF, postoperative heart failure; PMI, perioperative myocardial infarction (defined as Troponin T≥2000 ng/L on or after the third postoperative day); severe PPM, prosthesis-patient mismatch<0.65 cm^2^/m^2^.

### NT-proBNP and diastolic function

In both pre- and postoperative evaluations, higher NT-proBNP was significantly associated with high LV filling pressure compared with low LV filling pressure (Tables 1 and 2). Between preoperative measurement and 6-month follow-up, NT-proBNP was significantly reduced among patients with preoperative low LV filling pressure (320 ng/L [170–760 ng/L] vs. 270 ng/L [150–490 ng/L]; *P* = 0.04) and patients with preoperative high LV filling pressure (1100 ng/L [510–2800 ng/L] vs. 760 ng/L [380–1080 ng/L]; *P*<0.001) (Fig 1). Both preoperatively and postoperatively, we found a weak but significant correlation between high LV filling pressure and NT-proBNP, with the Spearman’s rank correlation coefficient between these variables being 0.32 (*P*<0.001) preoperatively, and 0.29 (*P*<0.001) postoperatively.

### LV systolic function and LV mass

Most patients with preoperative low LV filling pressure exhibited normal left ventricular systolic function, corresponding to an ejection fraction (EF) of >52% (n = 174; 90%). On the other hand, the proportion of patients with normal systolic function was significantly lower among patients with preoperative high LV filling pressure (n = 34; 63%; *P*<0.001). Preoperatively, the LV mass index (LVMI) tended to be higher in patients with preoperative high LV filling pressure compared to those with preoperative low LV filling pressure, although this difference did not reach significance (Table 1).

At the 6-month follow-up, LV mass had decreased significantly in both groups: from 125 g/m^2^ [101–146 g/m^2^] to 96 g/m^2^ [83–109 g/m^2^] (*P*<0.001) in the low LV filling pressure group, and from 137 g/m^2^ [109–172 g/m^2^] to 109 g/m^2^ [89–129 g/m^2^] (*P*<0.001) in the high LV filling pressure group. On the other hand, the LVMI at the 6-month follow-up was significantly higher in patients with preoperatively high LV filling pressure compared to those with preoperative low LV filling pressure (Table 2). LV mass index regression was similar between both groups (Table 2).

### Mortality

**Fig 2:**
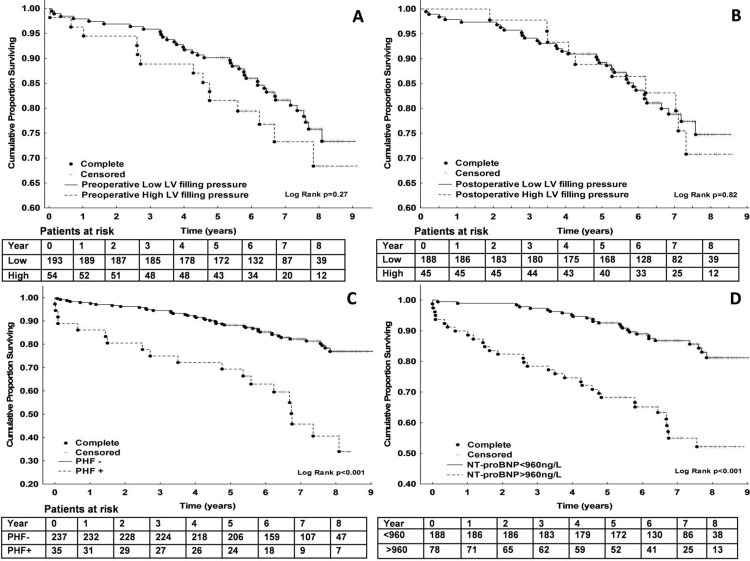
Kaplan-Meier survival curves categorized according to diastolic function before surgery (A), diastolic function at the 6-month postoperative follow-up (B), presence of PHF (C), and preoperative NT-proBNP above or below 960 ng/L (D).

Within the whole cohort, 30-day mortality was 1.1% (n = 3), 1-year mortality was 3.7% (n = 10), and 5-year mortality was 14% (n = 39). The median follow-up was 7.1 years (minimum, 4.1 years; maximum, 9.1 years), with a total of 63 deaths (23%). Long-term mortality did not significantly differ in relation to preoperative or postoperative LV filling pressure (Fig 2). ROC analysis revealed that a preoperative NT-proBNP of >960 ng/L was the best cut-off value for predicting all-cause 5-year mortality, with an area under the curve (AUC) of 0.7 (95% CI, 0.6–0.8; *P*<0.001). Fig 2 (panels C and D) shows the KaplanMeier plots of cumulative long-term survival according to PHF and NT-proBNP>960 ng/L. The final Cox regression analysis showed that long-term mortality was associated with diabetes with insulin or oral treatment, preoperative NT-proBNP>960 ng/L, renal dysfunction, and age, whereas female sex appeared to be protective (Table 6).

**Table 6:** Cox regression mortality, univariable and multivariable analyses.

Variable	Regression Coefficient (β)	SE	*P* value	RR	95% CI
Age, years	0.07	0.02	<0.001	1.07	1.03–1.10
Sex, male	Ref.				
Sex, female	−0.63	0.27	0.02	0.53	0.31–0.90
BMI	−0.003	0.03	0.92	1.00	0.94–1.06
P-Creatinine clearance, mL/min/m^2^	−0.02	−0.04	<0.001	1.00	0.96–0.99
CCT, min	0.01	0.006	0.08	1.01	1.00–1.02
ECC, min	0.009	0.003	0.003	1.01	1.00–1.01
NT-proBNP>960 ng/L	1.3	0.26	<0.001	3.59	2.15–6.0
DM	0.69	0.28	0.01	2.01	1.15–3.51
HT	0.44	0.27	0.09	1.56	0.93–2.62
Cardiac failure	1.01	0.29	<0.001	2.75	1.55–4.86
Concomitant CABG	0.69	0.26	0.008	1.99	1.20–3.30
Preoperative high filling pressure	0.35	0.31	0.26	1.42	0.77–2.63
Systolic function					
Mild—severe LV dysfunction	0.77	0.32	0.02	2.17	1.15–4.08
PHF	1.23	0.28	<0.001	3.44	1.98–5.95
PMI	1.33	0.43	0.002	3.76	1.62–8.74
**Final model**					
NT-proBNP>960 ng/L*	0.90	0.30	0.002	2.47	1.38–4.41
Age, years	0.06	0.02	0.004	1.06	1.02–1.10
DM, insulin or oral treatment	0.87	0.31	0.005	2.38	1.30–4.32
Female	−0.87	0.32	0.006	0.42	0.22–0.78

BMI, body mass index; CCT, cross-clamp time; ECC, extra corporeal circulation; DM, diabetes mellitus; HT, hypertension; CABG, coronary artery bypass surgery; PHF, postoperative heart failure; PMI, perioperative myocardial infarction (defined as Troponin T≥2000 ng/L on or after the third postoperative day); **, best cut-off point for preoperative NT-proBNP with regards to discriminating 5-year mortality.

## Discussion

The main findings of our present study were that a large proportion of patients with preoperative high LV filling pressures had normalized LV filling pressure at their 6-month postoperative follow-up. Despite this normalization, the overall proportion of patients with high LV filling pressure remained almost the same before and after surgery due to the LV deterioration in 12% (n = 24) of the patients with preoperative low LV filling pressure.

Notably, neither preoperative nor postoperative high LV filling pressure had a statistically significant effect on long-term survival. On the other hand, poor long-term survival was associated with both high preoperative NT-proBNP and PHF.

Preoperative high LV filling pressure was associated with PHF and longer ICU stay.

Both preoperative and postoperative high LV filling pressure were characterized by higher NT-proBNP levels, although both groups showed significantly decreased NT-proBNP at follow-up. Patients who changed from preoperative low LV filling pressure to postoperative high LV filling pressure group exhibited longer aortic cross-clamp times and cardiopulmonary bypass times, and a greater proportion had concomitant CABG procedures. These findings may suggest a higher degree of intraoperative ischemic injury, potentially leading to postoperative deterioration of diastolic function. This explanation is supported by the higher levels of CK-MB in this patient group, and is in line with previous research [16, 17].

We could not detect any impact of medication on the changes in LV filling pressures from preoperative state to postoperative state at six months, but a larger proportion of patients were on beta blockers in the cohort with high LV filling pressures preoperatively.

### Left ventricular diastolic function and NT-proBNP

Non-invasive evaluation of diastolic function is complex and must be based on several echocardiographic parameters. The American Society of Echocardiography (ASE) and the European Association of Cardiovascular Imaging (EACVI) recently provided updated recommendations to standardize diastolic function assessment [7]. Although the authors of these recommendations mention the age dependency of diastolic function parameters, this is not accounted for in their classification algorithms. Therefore, we have included age-adjusted reference values in a guideline-based decision support previously published by our group [14].

The relationship between NT-proBNP and diastolic function has been extensively studied [18–22]. In line with prior findings, we also identified an association between diastolic function and NT-proBNP. After adjustment for common confounders, high preoperative NTproBNP was the sole predictor for preoperative high LV filling pressure.

One interesting and, to our knowledge, novel finding was that among patients with preoperative high LV filling pressure, those who exhibited improvement at the 6-month follow-up showed significantly higher levels of preoperative NT-proBNP and a higher LVmass index compared to patients who remained in the high LV filling pressure group, although both groups showed similar proportions of left ventricular hypertrophy. The group without improvement also exhibited significantly higher proportions of patients with diabetes mellitus and with postoperative atrial fibrillation.

Knowledge about the connection between AS, DM, NT-proBNP, and progression to fibrosis is currently expanding [23, 24]. Our present findings might suggest that patients without improvement at 6 months postoperatively had a more fixed diastolic dysfunction due to fibrosis; however, this possibility was not investigated in our study. Hypothetically, this could explain the difference in NT-proBNP levels, assuming that a more fibrotic LV releases less NT-proBNP than an LV wall containing more cardiac muscle cells and a lower proportion of fibrosis. On the other hand, a study of a community-based cohort recently reported that elevated NT-proBNP levels are associated with diffuse myocardial fibrosis [25]. However, in AS patients, myocardial fibrosis predominantly takes the form of diffuse interstitial fibrosis early in the disease process and might progress to replacement fibrosis with scarring and myocyte loss at end-stage heart failure [24].

### Mortality and postoperative heart failure

The present results confirm our previous observations that PHF is associated with poor long-term survival, although it may initially appear benign [26]. Based on our previous study, we proposed that an episode of PHF may unmask a myocardial factor responsible for the delayed consequences. Since our previous study included adjustment for systolic function, it was reasonable to suggest that diastolic dysfunction might be involved [10]. The presently identified association between preoperative NT-proBNP and poor long-term survival, and our Cox regression model, support the assumption that an underlying myocardial factor is responsible. However, the current results do not clearly identify diastolic dysfunction as the culprit, since high LV filling pressure was not associated with increased mortality. Notably, this does not exclude that diastolic dysfunction due to fibrosis may be important for long-term outcome since the utilized methodology cannot discriminate between high diastolic LV pressure caused by hypertrophy versus fibrosis.

The literature includes conflicting data regarding the role of diastolic function for prognosis after AVR for AS [27, 28]. As mentioned earlier, different generations of guidelines have recommended different ways of classifying diastolic function. It has also been demonstrated that even when authors refer to the use of a certain classification, they may still apply different priorities and modes of action that influence the results [29]. There can also be differences in referral patterns, such that in some cohorts, the average patient might be considered for surgery at a later point in the disease process, and thus be in a more advanced state in terms of heart dysfunction. Other factors to consider are the differences in cohort sizes and outcome measures [30]. The missing link could be the degree of fibrosis. Future studies are needed to clarify the coupling between degree of fibrosis, improvement in diastolic function, and outcome after SAVR.

Although our present study population was relatively large, one major limitation was the low number of events. Missing data and excluded patients due to atrial fibrillation or MAC (Mitral annular calcification) contributed to this limitation. We were ultimately able to evaluate time-to-event in 52 of our 247 patients, and thus we must consider the possibility of a type II error. However, it is apparent that variables other than diastolic dysfunction were of greater importance in predicting outcome. Another limitation of our study is that when addressing long-term mortality, we did not differentiate between cardiac mortality and mortality due to other causes.

To conclude, our key points are SAVR improved diastolic function in patients with aortic stenosis and high LV filling pressure in 50% of the patients. Our results could not confirm the previously suggested role of diastolic dysfunction as a marker for poor long-term survival after SAVR. Our results indicate that both high preoperative NT-proBNP levels and an episode of postoperative heart failure after SAVR were associated with impaired long-term survival, in agreement with previous studies.
